# Masseter muscle rigidity: Atypical malignant hyperthermia presentation or isolated event?

**DOI:** 10.4103/1658-354X.71580

**Published:** 2010

**Authors:** Tonia C. U. Onyeka

**Affiliations:** *Department of Anaesthesia, University of Nigeria Teaching Hospital, Ituku-Ozalla, Enugu, Nigeria*

**Keywords:** *Atypical malignant hyperthermia*, *intubation*, *laryngoscopy*, *laryngeal mask airway*, *masseter muscle rigidity*, *suxamethonium*

## Abstract

This report describes a case of masseter muscle rigidity encountered at the start of an elective gynaecological procedure. At preoperative assessment, the patient, a 41-year old woman with a previous non-eventful surgical and anesthetic history was given a Mallampati score of 3. Following suxamethonium administration, full mouth opening proved difficult. Laryngoscopy and tracheal intubation were not possible leading to the eventual use of a laryngeal mask airway and resulting in a successful anaesthetic outcome. A number of possibilities that may account for this situation as well as viable options for airway access in such cases are discussed below.

## INTRODUCTION

Suxamethonium is a depolarizing neuromuscular blocker, widely available for use during endotracheal intubation because of its rapid onset of muscle relaxation. However, one known adverse effect of this drug is masseter muscle rigidity (MMR) sometimes referred to as masseter muscle spasm. This case report highlights the possibility of MMR existing in isolation. It also shows the need to have malignant hyperthermia (MH) and other differentials like temporomandibular joint (TMJ) dysfunction uppermost on one’s mind when such difficulty is encountered. Finally, it points to the possibility of successfully using a laryngeal mask airway (LMA) when endotracheal intubation fails in such cases.

## CASE REPORT

A 41-year-old female with menstrual irregularities was booked for a laparotomy and salpingooophorectomy. She had no relevant medical history and had an uneventful general anesthesia for a cesarean section nine years ago. She weighed 65 kg, had normal vital signs, adequate mouth opening, and was assigned a Mallampati score of 3 and American Society of Anesthesiologist (ASA) physical status of 1.

In theatre, equipments for difficult intubation were assembled and patient was connected to standard monitors. Following premedication with intravenous atropine 0.6 mg, anesthesia was induced with fentanyl 100 μg and of ketamine 100 mg. Intravenous suxamthonium 100 mg was given to facilitate endotracheal intubation. No muscle fasciculation was observed after several minutes following suxamethonium. The first attempt at laryngoscopy using a size 4 Mac laryngoscope blade was then made but proved difficult as the patient’s mouth was unable to open fully to allow advancement of the laryngoscope. Patient was mask ventilated while the suxamethonium vial was reexamined to check for its shelf life. Next, 6 mg of pancuronium was administered and after waiting a full 3 minutes to allow the nondepolarising neuromuscular agent take effect, the second and third attempts were made. But the difficulty in advancing the laryngoscope fully into the mouth to be able to visualize the larynx persisted. The next decision taken immediately was to insert a size 4 laryngeal mask airway (LMA), which was successful and the rest of the surgery was conducted uneventfully.

## DISCUSSION

The so-called “Jaws of steel” which many anesthetists have alluded to masseter muscle rigidity (MMR) in the past[1] usually manifests as an increase in the tension in jaw muscles.[2] Malignant hyperthermia (MH), a hypermetabolic reaction to suxamethonium, vecuronium,[3] inhalational agents, and occasionally stress, has a wide and varied range of clinical presentation that frequently makes diagnosis both challenging and difficult. Muscle rigidity as seen in MMR has been said to be an early indicator of MH.[4] Its incidence is less than 1% in children,[5] while the incidence in adults is unknown.[6] In pediatric patients, MMR can result if patient is under dosed (<1 mg/kg intravenous succinylcholine) or if myotonic syndrome is present.[5] Some believe that isolated MMR is not pathognomonic of MH.[4] This patient had no problems with general anesthesia in the past and had no relatives who had problems previously under general anesthesia during surgery. No truncal rigidity was observed and her vital signs remained stable.

Suxamethonium is the only depolarizing agent in use in our institution for endotracheal intubation. Its features include fasciculation, histamine release, hyperkalaemia, dysrrythmias, and malignant hyperthermia amongst others. Suxamethonium can increase the tone of the masseter muscles, a response that is transient and maximal at the end of facial fasciculations.[7] However, in susceptible patients this suxamethonium-induced increase in muscle tone can progress to MMR.[8] It has been suggested that when used as an induction agent, propofol reduces the masseter muscle tension more effectively than sodium thiopentone.[7] One may consider that an opioid, fentanyl was administered at induction. But high doses of opioids are more commonly known to produce rigidity at induction in the nonparalyzed patient[9] than low doses, with the occasional instance where 150 mcg of fentanyl produced rigidity in a patient on concomitant haloperidol.[10]

The possible causes of the difficult laryngoscopy, difficult intubation and the consequent failed intubation encountered in this situation were considered. Difficult laryngoscopy is known to occur in 1.5 to 8% of all general anesthetics[11] and the Mallampati test is sometimes used preoperatively to predict the ease or difficulty of airway access. But some studies show that the Mallampati tests have limited accuracy, and are therefore not suitable screening tools for the difficult airway.[11] Although this patient’s Mallampati score of 3 pointed to a potentially difficult airway, it was not considered it a significant predictor of this event. In some instance, unsuspected temporomandibular joint (TMJ) dysfunction had been mistaken for MMR.[12] This possibility was ruled out in this case by the absence of inability to open the mouth to >1 cm and the absence of firmly approximated teeth.

Failed intubation is an important cause of morbidity and mortality during anesthesia and requires that the anesthetist uses an alternative technique to secure the airway, especially in cases requiring rapid sequence induction (RSI). Retrograde endotracheal intubation (REI),[8] asleep fibreoptic intubation with spontaneous respiration after recovery from suxamethonium, and blind nasotracheal intubation are viable options. Surgical cricothyroidotomy has been employed in a case difficult intubation following MMR with success[4] while in another due to suxamethonium-induced TMJ subluxation, a Trachlight™was employed.[13] A LMA was used successfully in this case and the latter’s safety in patients with the difficult airway and without any of its contraindications is well documented.[14]

In summary, this case is one of difficult laryngoscopy and difficult intubation from masseter muscle tension, resulting in failed intubation and use of LMA for airway maintenance. All possibilities, ranging from MMR (suxamethonium-induced or as a sign of MH) to subluxation of TMJ should be ruled out and the airway secured promptly through any convenient means to avoid the morbidity and mortality associated with the difficult airway. In addition, such patients should have a muscle biopsy for caffeine test done to rule out malignant hyperthermia and should be informed of the incident for future medical procedures on themselves and their offspring too.
